# A Multivariate Methodological Workflow for the Analysis of FTIR Chemical Mapping Applied on Historic Paint Stratigraphies

**DOI:** 10.1155/2017/4938145

**Published:** 2017-12-03

**Authors:** Giorgia Sciutto, Paolo Oliveri, Silvia Prati, Emilio Catelli, Irene Bonacini, Rocco Mazzeo

**Affiliations:** ^1^Department of Chemistry, Microchemistry and Microscopy Art Diagnostic Laboratory (M2ADL), University of Bologna, Via Guaccimanni 42, 48100 Ravenna, Italy; ^2^Department of Pharmacy, University of Genova, Viale Cembrano 4, 16148 Genova, Italy

## Abstract

In the field of applied researches in heritage science, the use of multivariate approach is still quite limited and often chemometric results obtained are often underinterpreted. Within this scenario, the present paper is aimed at disseminating the use of suitable multivariate methodologies and proposes a procedural workflow applied on a representative group of case studies, of considerable importance for conservation purposes, as a sort of guideline on the processing and on the interpretation of this FTIR data. Initially, principal component analysis (PCA) is performed and the score values are converted into chemical maps. Successively, the brushing approach is applied, demonstrating its usefulness for a deep understanding of the relationships between the multivariate map and PC score space, as well as for the identification of the spectral bands mainly involved in the definition of each area localised within the score maps.

## 1. Introduction

Over the last decade, many research studies were focused on the development of advanced analytical methods for the characterisation of heterogeneous materials and their stratigraphic localisation in paint cross-sections.

FTIR micro-spectroscopy was widely applied to the characterisation of heritage materials [1–5], and it still represents one of the most popular approaches in the field of conservation science, as it allows the simultaneous detection of both organic and inorganic substances, as well as their localisation within complex sample matrices. Moreover, in the last decades, FTIR microscopy potentialities were increased thanks to the introduction of mapping and imaging devices, which allow collecting a large number of FTIR spectra from a sample area, by applying different modes of analysis, such as total reflection and attenuated total reflection (ATR) [5–7].

FTIR chemical maps/images can be obtained plotting the intensities of characteristic absorption bands, coded by a chromatic scale, in correspondence with their spatial position on a selected sample area. However, if a univariate study is performed on the complex matrix of spectral data (hyperspectral cube), a straightforward interpretation of the results is usually difficult, as it can be affected by matrix effects, presence of mixtures, the overlapping of characteristic bands, and changes in their relative intensities. Thus, it has been widely demonstrated that when data sets are constituted by multiple objects and variables, univariate methods—which examine one variable at a time—considerably underutilise the information enclosed therein. Conversely, multivariate approaches are able to consider and represent the whole information in an easily understandable way [8–10].

Therefore, it is usually profitable to perform multivariate analysis in order to extract the maximum useful information embodied in the spectral dataset. Principal component analysis (PCA)—which is one of the most common and efficient tools for multivariate data exploration—can be performed, after application of a proper signal preprocessing [8, 9]. The score values can be used to build up bidimensional scatter plots (the score plots), representing the projections of the data objects into a Cartesian space defined by two given Principal Component axes. A score plot gives information about the multidimensional structures existing among the objects, such as similarities, groupings, and trend patterns. Another possibility for representing the object score values is to use a chromatic scale, for example, from blue (minimum) to red (maximum score value), recreating a 2D false colour map.

However, even though a small number of applications of such an approach have been reported so far [11–15], in many cases chemometric tools are underutilised and the outcomes underinterpreted. Indeed, the simple creation of score maps and the visualisation of score scatter plots and loading profiles may lead to the uncorrected interpretation of chemometric results and to the loss of information.

Within this scenario, the present work was aimed at disseminating the usage of methodologies—which are well-established in the chemometric field—within the field of applied researches in heritage science, in which the use of multivariate methods is still quite limited. To this aim, a representative group of case studies, on which a chemometric approach was successfully applied in practice, is presented. This is intended to provide the reader with an illustrative guide, to better establish the use of chemometric tools in the interpretation of complex data sets related to the investigation on paint cross-sections.

In the methodological workflow proposed, the two approaches for the representation of scores—namely, scatter plots and score colour maps—are used in a comprehensive strategy (brushing approach), allowing an exhaustive interpretation of chemometric results. In more detail, brushing approach is a key tool for deeply understanding relationships between the image space and the PC score space. Clusters of scores present within the scatter plot can be selected and localised within a PC score map, by highlighting of the correspondent objects. Thus, the brushing approach permits the unambiguous identification of the investigated area corresponding to each score cluster [16, 17].

Furthermore, the spectral profiles of such objects can be extracted and interpreted, jointly with the analysis of the loading values, which is functional to individuate the spectral bands that are most involved in defining each subarea under investigation. Finally, PC false colour images (RGB) can be obtained by coding the score values on three selected PCs as the intensity of the red, green, and blue channels. This generally reveals the distribution of the various painting materials in a single image.

The aim of this paper is to present practical examples in which chemometric studies helped in the selection of proper restoration practice, demonstrating the utility of the method that could be applied to support routine investigation.

The three exemplificative cases were chosen to embrace different conservation issues relate to the characterisation of the execution technique and the material applied during past restoration campaign to support ongoing conservation actions. Moreover, the multivariate approach was applied for all the samples for the identification and localisation of thin organic layers ascribable to finishing (such as varnishes) or preparation layers. The identification of such thin layers still represents a challenging task in the field of conservation science not only for their limited thickness but also for their complex chemical compositions. In more detail, the first case of study (sample 15r1) was referred to a recent study on a painting on paper considered one of the most important example of mosaic replica in Italy. For the first time, such a type of artistic sample has been investigated by analytical methodologies, revealing new insights on the execution technique adopted. In addition, a sample was collected from a mural painting (XI century), characterised by the presence of surface treatments applied during past restoration interventions (sample PO1). A practical example in which chemometric studies helped the selection of proper restoration practice was reported, demonstrating the utility of the method that could be applied to support routine investigation. Finally, the paper showed the results obtained on a sample collected from a rare example of ancient celestial globe dated back to the XVII century (sample BC1). Interestingly, the present research showed the potentialities of a multivariate approach, which allowed obtaining more information than what was previously obtained with the use of univariate method and revealing the presence of a thin proteinaceous layer.

## 2. Materials and Methods

### 2.1. Samples

Three paint samples characterised by the presence of different types of organic coatings/treatments were submitted to *μ*ATR-FTIR analyses, and their chemical composition and stratigraphic spatial location were determined by means of a multivariate approach.

**Figure 1:**
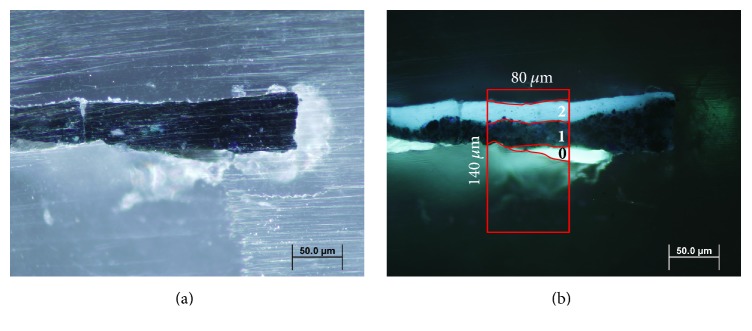
Cross-section microphotographs of sample 15r1 embedded in KBr. (a) Image under visible light; (b) image under UV illumination; the red box indicates the selected area for *μ*FTIR-ATR analysis.

The first sample (sample 15r1) was investigated in the framework of a project aimed at archiving and preserving a wide collection of mosaic replicas (referred to as* “cartoni musivi”* in Italian), painted in Ravenna between the second half of the XIX century and the first decades of the XX century. They were mainly used as a documentation tool during mosaic restoration workshops. The sample analysed was collected from a blue painted area of the mosaic paper replica* The Doves* (1940), by Alessandro Azzaroni (Figure 1(a)).

The second sample (sample PO1) was collected from the lower part of the west wall of the XI century mural paintings located in the Pomposa Abbey (Codigoro, Ferrara, Northern Italy).

The third sample (sample BC1) was sampled from the wooden support of a celestial globe executed by Vincenzo Coronelli in the XVII century.

### 2.2. Sample Preparation

Paint fragments were embedded in potassium bromide (KBr, purity > 99.9%, by Sigma Aldrich Inc, USA) and cross-sectioned by following a standardised procedure which includes a dry polishing on Micro-Mesh® silicon carbide papers (Micro-Surface Finishing Inc, Wilton, USA) with successive grid from 2400 up to 12,000 [18].

### 2.3. Optical Microscopy

Sample cross-sections were primarily observed under optical microscopy in order to document the stratigraphic morphology of the paint layers. A dark field observation was performed, with a BX51 (Olympus Optical, Tokyo, Japan) microscope equipped with a digital scanner camera Olympus DP70. A 100-W halogen projection lamp and a Ushio Electric USH102D ultraviolet (UV) lamp (Ushio Inc, Tokyo, Japan) were employed for the acquisition of visible and fluorescent images, respectively.

### 2.4. *μ*ATR-FTIR Analysis

A Thermo Nicolet iN™10MX imaging microscope (Thermo Fisher Scientific, Waltham, MA, USA), fitted with a mercury-cadmium-telluride (MCT) detector cooled by liquid nitrogen, was used for mapping analysis. Measurements were performed using a slide-on ATR objective, equipped with a conical germanium crystal, in the range 4000–675 cm^−1^, at a spectral resolution of 4 cm^−1^. Backgrounds were acquired keeping the slide-on inserted and the ATR objective not in contact with the sample surface.

For ATR mapping on sample 15r1, an area of 140 × 80 *μ*m^2^ was analysed, with a step of 10 *μ*m in the *x*-*y* direction and an aperture of 60 × 60 *μ*m, corresponding to an investigated area of about 15 *μ*m^2^ for each single point analysed. A total of 180 spectra were recorded.

Sample PO1 was mapped using an aperture of 30 × 30 *μ*m (with an effective investigated area of about 7.5 *μ*m^2^ for each point of analysis) and a step of 4 *μ*m in the *x*-*y* direction. The overall area of analysis was of 92 × 84 *μ*m^2^, with 528 spectra recorded.

A Nicolet Nexus 5700 spectrometer combined with a Thermo Continuum IR microscope (Thermo Fisher Scientific, Waltham, MA, USA) was employed for the analysis of sample BC1. The infrared spectra were measured in the spectral range 4000–650 cm^−1^ at a spectral resolution of 4 cm^−1^. A micro-slide-on ATR silicon crystal, directly connected to the microscope objective, was used to collect a total of 192 spectra on a selected area of 300 × 220 *μ*m^2^. The selected aperture was 150 × 150 *μ*m (effective investigated area of approximately 40 *μ*m^2^) and the step size was 20 *μ*m in the *x*-*y* direction.

The dedicated software OMNIC™ and OMNIC Picta™ (Thermo Fisher Scientific, Waltham, MA, USA) were used for the preliminary manipulation of the overall spectra dataset.

### 2.5. Multivariate Chemical Mapping

Multivariate data processing and chemical mapping were performed by means of in-house MATLAB routines (The Mathworks Inc., Natick, USA). Suitable row pretreatments were selected and applied in attempt to minimise systematic unwanted variations, which could affect the signals. In more detail, for samples 15r1 and PO1, a linear detrending was applied, while the standard normal variate (SNV) transform was selected for sample BC1. In particular, linear detrending removes the best straight-line fit from a signal and, therefore, it corrects for linear baseline drift, which often represents a typical issue [19]. The standard normal variate (SNV) transform, or row autoscaling, is a pretreatment particularly used for spectral data, which allows correcting for both baseline shifts and global intensity variations [20]. Afterwards, column autoscaling was performed for sample 15r1 and column centring was performed for both samples PO1 and BC1. Spectral regions were reduced, eliminating those infrared parts affected by environmental and instrumental noise that prevalently embody unhelpful information. In particular, the spectral range between 2200 and 2400 cm^−1^ (characterised by the presence of the CO_2_ absorption bands) and the terminal parts of the spectral range (4000–3900 cm^−1^; 675–700 cm^−1^) were removed.

PCA was performed using the nonlinear iterative partial least squares (NIPALS) algorithm, which allows stopping the computation of principal components either at the desired number or at a predetermined level of cumulative explained variance. After PC computation, scores were obtained for each grid point of the map and were used both for drawing scatter score plots (in a Cartesian plane defined by a couple of PCs) and for reconstructing score maps (in which scores are refolded in the map grid and coded by a given colour scale). To understand the relationships among groupings of points in the score scatter plot and spatial regions in the score map, the so-called brushing procedure is applied. Typically, brushing is performed by selecting a cluster of points of interest within the scatter plot: the software automatically recognises the corresponding points in the map grid and graphically highlights the resulting region in the map. The procedure can also be performed in the opposite direction, by selecting spatial regions within the score map and automatically highlighting the corresponding points in the score scatter plot. The brushing approach is particularly useful, from an exploratory point of view, since it allows not only understanding relationships between areas in the score map and clusters in the score scatter plot, but also determining the contribution of the spectral variables in characterising each region in the map. This last achievement is possible by jointly analysing score maps, score scatter plots, and score loading plots—in which the importance of the original spectral variables in the definition of two given PCs is shown. Loading values also give information about intercorrelation among variables (the closer the loading values, the higher the correlation degree between a couple of variables) and can be also represented as intensity profiles against the spectral variables. Such a representation allows establishing a direct parallelism between spectral regions revealed as important by loading values and original absorption bands in spectral profiles.

All the steps of the data processing described were performed by means of in-house MATLAB (The MathWorks, Inc.) routines, which are freely available from the authors upon request, as well as the datasets presented in the present paper.

## 3. Results and Discussion

### 3.1. Sample 15r1

The sample was investigated in the framework of a project aimed at archiving, preserving, and valorising a wide collection of mosaics replicas. Taking this opportunity, a research work focused on the evaluation of state of conservation as well as on the characterisation of painting materials and techniques was implemented.

Microscope observations of sample 15r1 show a simple stratigraphy, better documented under UV illumination, which highlights the presence of three different layers (see Figures 1(a) and 1(b)). In particular, the support made of paper (layer 0, 18 *μ*m thick) presents a strong white fluorescence, while the superimposed blue pigment layer is characterised by a homogeneous dark matrix with small dispersed particles (layer 1, 27 *μ*m thick). The uppermost layer (layer 2, 20 *μ*m thick)—which is not clearly identifiable under visible light—shows a bright bluish fluorescence that can be conceivably assigned to the application of a protective/finishing coating. *μ*ATR-FTIR measurements were performed on a representative sample area (red box in Figure 1(b)).

**Figure 2:**
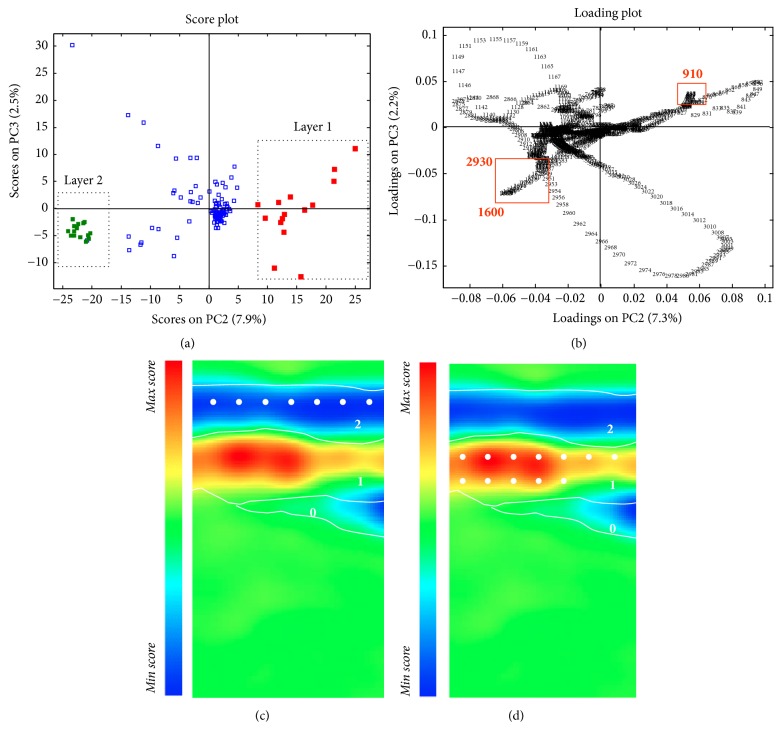
Sample 15r1. (a) PC23 score plot: clusters highlighted in red indicate the objects points localised within layer 2 (white dots in (c)) and green for points localised within layer 1 (white dots in (d)); (b) PC23 loading plot; (c, d) PC2 score map.

A multivariate exploratory study was performed in order to extract the maximum relevant information from the overall set of spectra recorded from the selected small area of analysis and to differentiate organic and inorganic substances. Among the three lowest-order principal components (Figure 2), the score map of PC2 (Figure 2(c)) presents the most interesting features that allowed the localisation of the materials constituting the three layers on the basis of their chemical nature, despite the weak spectral differences observable.

**Figure 3:**
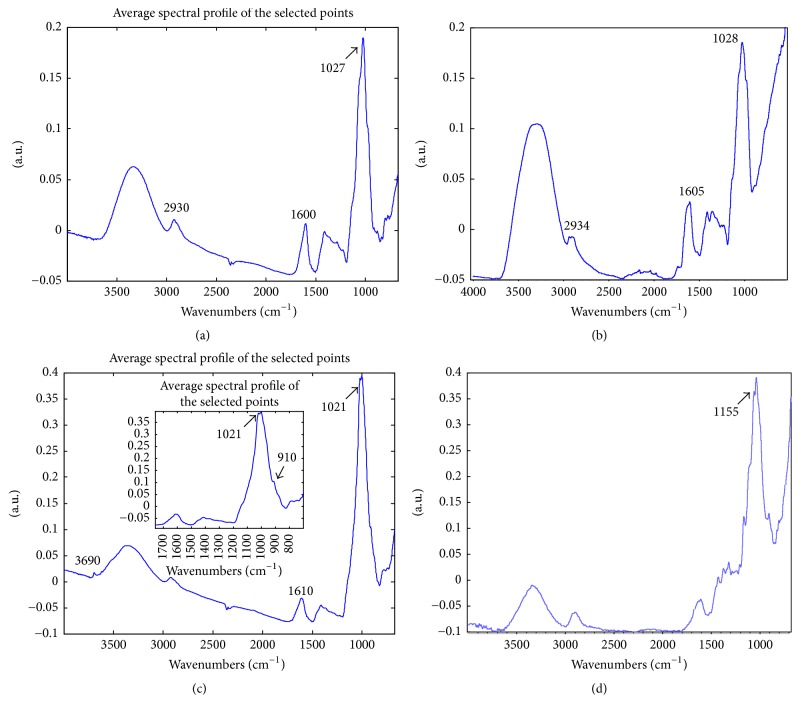
Sample 15r1. (a) Average spectral profile extracted from layer 2; (b) reference spectrum of a natural gum (peach gum purchased by Zecchi, Florence Italy); (c) average spectral profile extracted from layer 1; (d) average spectral profile extracted from layer 0.

After the preliminary map analysis, score and loading plots (Figures 2(a) and 2(b)) were examined, in order to identify the spectral bands that mainly characterise each layer and to better evaluate the information enclosed within multivariate maps. In particular, in the PC2-3 score plot (Figure 2(a)), two different groups of points are shown, related to high (red squares) and low (green squares) score values along PC2. These clusters correspond to layers 1 and 2 visualised in the PC2 score map, respectively (Figures 3(c) and 3(d)). The correspondence with the loadings located in the matching directions reveals that a crucial role in describing layer 2 is played by a well-defined band at 1600 cm^−1^ (O–H groups) and the spectral band at 2930 cm^−1^ (C–H aliphatic stretching absorption bands). This information was supported by the examination of the average profile of the spectra collected from layer 2 (Figures 3(a) and 3(b)), which was characterised by the simultaneous presence of an intense absorption band at 1027 cm^−1^, suggesting the presence of a polysaccharide-based material, probably a coating made of a natural gum.

On the contrary, the red area—corresponding to layer 1 (see Figure 2(d))—describes the well localised blue paint layer. According to loading analysis, the examination of average extracted spectra allows the identification of a band at 3690 cm^−1^ (O–H stretching of hydrated silicate), probably linked to the presence of kaolin mixed with ultramarine blue, together with the band at 910 cm^−1^ (Si–O–H stretching) which helps in the discrimination of layer 1 as revealed by the loadings analysis (Figure 3(c)). More challenging was the clear identification of the binding media used in such pigment layer, because the presence of inorganic pigment could hamper the detection of the usually very limited amount of organic binder. In the specific sample, it was possible to hypothesise the presence of polysaccharide material used as binder. However, it is worth noticing that, in different areas, lipidic substances have been also identified. This is consistent with the fact that these copies were realised by artists (typically restores) for documentation purposes, and they needed and were asked to obtain specific effects in order to reproduce the original mosaic. Thus, the use of different pigment mixtures or different binding media, even in the same painting, may be required.

Finally, the layer 0 was characterised by the presence of a band at 1155 cm^−1^ ascribable to C–O–C antisymmetric stretching of cellulose (Figure 3(d)).

**Figure 4:**
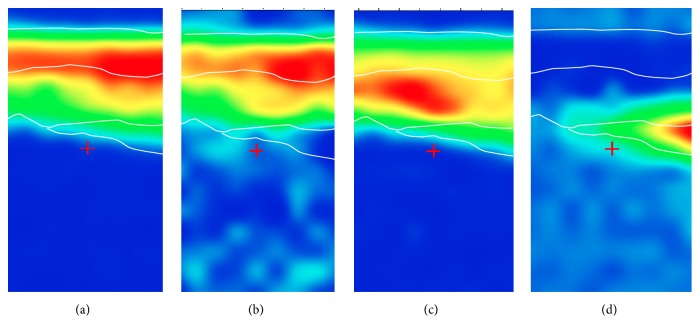
Sample 15r1. Univariate chemical maps obtained by integration of the absorption band at (a) 1600 cm^−1^; (b) 2928 cm^−1^; (c) 1030 cm^−1^; (d) 1155 cm^−1^.

For comparison, univariate approach was also applied. Nevertheless, due to the similar spectral features of components present in the stratigraphy, the univariate method, which is based on the intensity of single bands, made it difficult to have a clear distinction between the layers (Figure 4).

### 3.2. Sample PO1

The diagnostic campaign performed on the mural painting of the Pomposa Abbey represented a fundamental step in the set-up of a pilot restoration intervention aimed at the requalification of the entire monument. Indeed, due to a very poor state of conservation, an urgent restoration was needed. Thus, in attempt to define the most suitable conservative interventions, a proper analytical protocol was adopted to characterise original and restoration materials, as well as the painting technique.

For sample PO1, the study was aimed at characterising the nature and stratigraphic localisation of surface treatments applied during past restoration interventions. Indeed, due to the strong absorption bands of the matrix of calcium carbonate, difficulties in the proper characterisation of the chemical nature of the varnish occurred.

**Figure 5:**
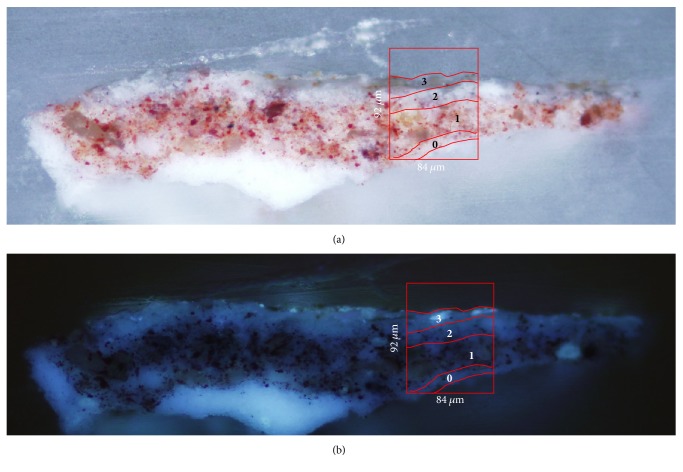
Cross-section microphotographs of sample PO1 embedded in KBr. (a) image under visible light; (b) image under UV illumination. The red box indicates the selected area for *μ*FTIR-ATR analysis.

Optical microscope observations (see Figure 5) revealed the presence of a white ground layer (layer 0) over which a paint layer with red pigment particles dispersed within a white matrix can be identified (layer 1, 52 *μ*m thick and layer 2, 17 *μ*m thick). Furthermore, a thin brownish external layer (layer 3, 10 *μ*m thick), which exhibits a whitish fluorescence under UV illumination (Figure 5(b)), can be documented in the stratigraphy.

**Figure 6:**
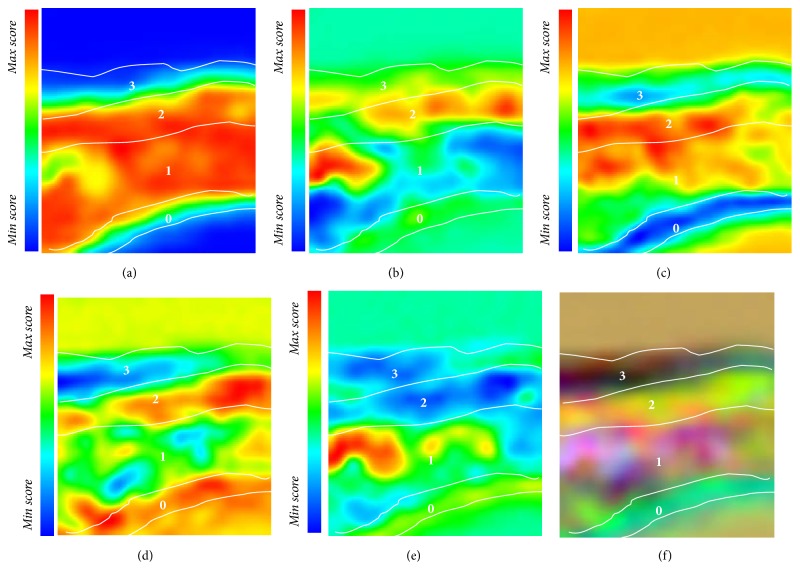
Sample PO1—score maps. (a) PC1, (b) PC2, (c) PC3, (d) PC4, (e) PC5, and (f) PC false colour image (RGB) obtained by coding the PC3, PC4, and PC5 score values as the intensity of the red, green, and blue channels, respectively.

*μ*ATR-FTIR measurements were performed on a selected area showed as a red box in Figures 4(a) and 4(b), and the resulting spectral datasets were submitted to PCA. Five PC score maps were taken into consideration for the interpretation of the entire paint stratigraphy (Figures 6(a)–6(e)).

**Figure 7:**
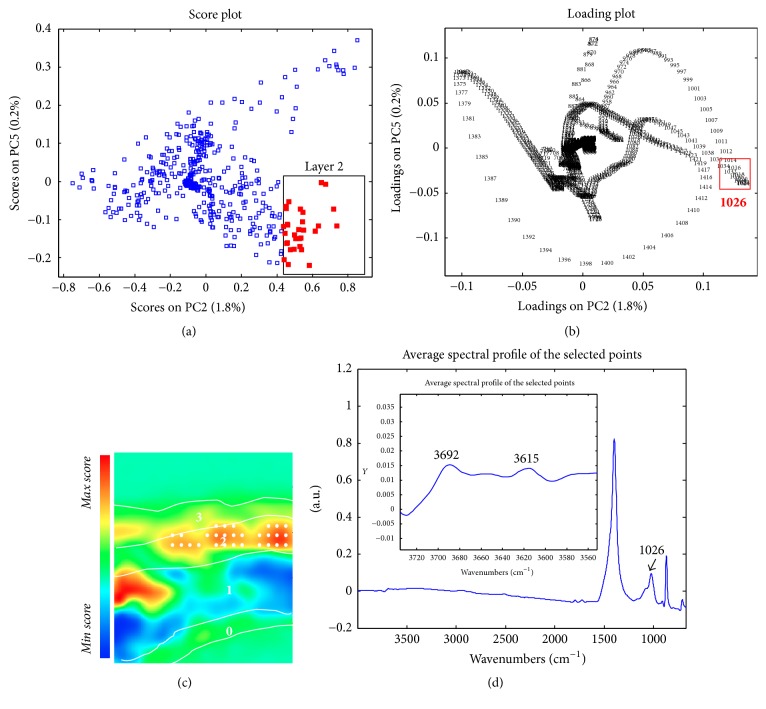
Sample PO1. (a) PC25 score plot: cluster highlighted in red indicate the objects localised within the PC2 score map (layer 1); (b) PC25 loading plot; (c) PC2 score map: The white points indicate objects localised within layer 1; (d) average spectral profile extracted from layer 2.

The average spectrum of layer 0, well recognisable in the PC3 score map, reported the presence of calcium carbonate as the main component* (data not shown)*. Differentiation between layers 1 and 2 is observable in the PC2 score maps. Indeed, layer 1 appeared to be characterised by the presence of calcium carbonate* (data not shown)*, while the joint analysis of scores and loadings (Figures 7(a) and 7(b)—PC2-5 score and loadings plot) allowed the identification of the higher contribution of the spectral variable at 1026 cm^−1^ (Si–O stretching) in the description of layer 2. The corresponding average spectral profile (Figure 4(d)) revealed the simultaneous presence of absorption bands at 3692 and 3615 cm^−1^ (O–H stretching), suggesting the presence of a hydrated silicate based material dispersed within a calcium carbonate matrix.

**Figure 8:**
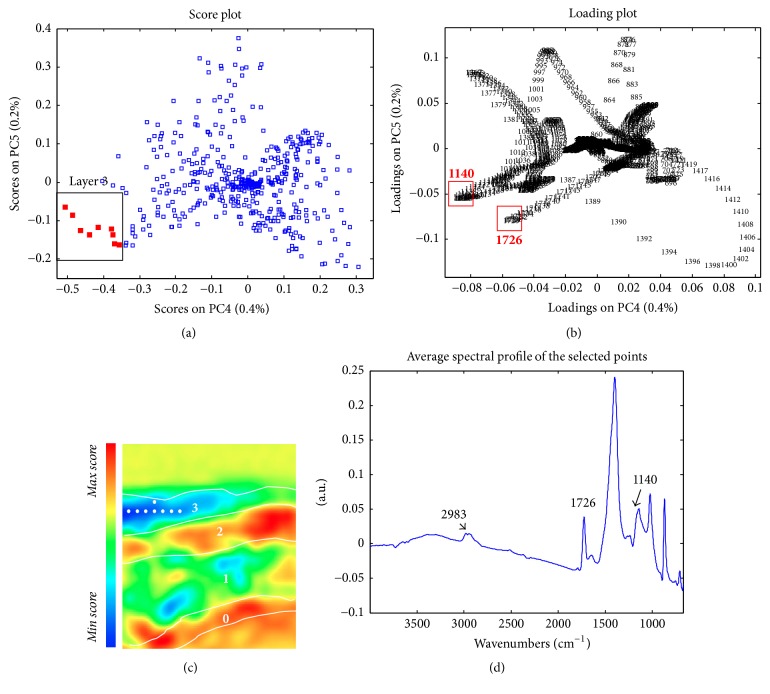
Sample PO1—(a) PC45 score plot: cluster highlighted in red indicates the objects localised within the PC4 score map (layer 3); (b) PC45 loading plot; (c) PC4 score map; (d) average spectral profile extracted from layer 3.

Apart from the inorganic components, a particular attention was devoted to the characterisation of the brownish external layer 3. Differentiation of such a layer from the others is visible in PC4 score maps (Figure 8(c)), thanks to the crucial role played by the spectral variable at 1726 cm^−1^, which is ascribable to the characteristic ester C=O stretching vibration of a synthetic resin. Moreover, the band at 1140 cm^−1^ (C–O–C stretching) contributes in chemically differentiating this layer from the others. The average spectral profile (Figure 8(d)) allowed the identification and spatial localisation of a very thin layer made of an acrylic-based varnish, which was applied during past restoration interventions. However, it is worth pointing out that no specific signal related to the presence of organic materials used as binders in layer 1 was found. This evidence may be linked either to the absence of such components or to their presence in a very low amount, whose identification within a complex matrix was quite challenging.

The PC false colour image (RGB) (Figure 6(f)) was obtained by coding the PC3, PC4, and PC5 score values as the intensity of the red, green, and blue channels. They were ascribed, respectively, to the presence of layer 0 (calcium carbonate), layer 1 (calcium carbonate and silicates), layer 2 (a hydrated silicate based material), and layer 3 (acrylic varnish), respectively.

### 3.3. Sample BC1

**Figure 9:**
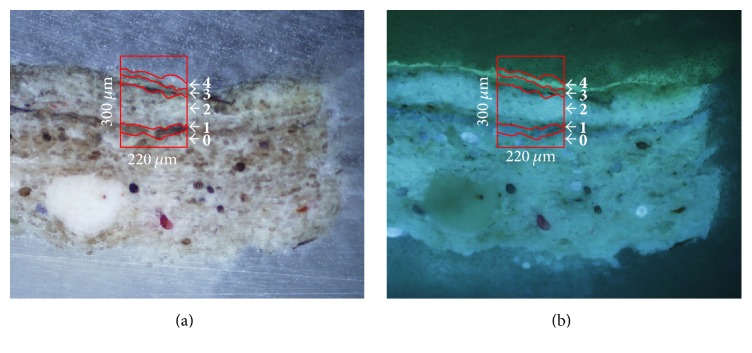
Cross-section microphotographs of sample BC1 embedded in KBr, (a) image under visible light; (b) image under UV illumination. The red box indicates the selected area for *μ*FTIR-ATR mapping.

The hyperspectral data cube of sample BC1, acquired and analysed during a former diagnostic campaign [6], was submitted to multivariate analysis aimed at extracting useful and new information, overcoming problems related to poor spectral and spatial quality, mainly induced by the use of an old ATR system. The area of sample BC1 submitted to the analysis is indicated with a red box in Figure 9.

A detailed description of sample stratigraphy was already reported in the previous study [6]. Briefly, the sample is characterised by the presence of two different layers of paper on the top of a wooden support (Figure 9, layers 0 and 2). The presence of an organic component, used in order to make paper resistant to water, is well recognisable in the UV fluorescence microphotography, thanks to the peculiar visible emission in layer 1 and layer 4 (15 *μ*m thick) (Figure 9(b)). The previous study, based on a univariate approach, allowed the identification and localisation of the two layers of cellulose by integration of the diagnostic band at around 1021 cm^−1^ (C–O stretching). The results showed also the presence of gypsum in layer 3. The authors linked such evidence to the sampling area, which was treated with gypsum to fill the gap induced by paper detachment in a previous restoration. On the other hand, even if it was possible to hypothesise the presence of a natural resin in layer 4 (shellac) as coating (according to the technical background [21]), it was not possible to clearly distinguish it from layer 1 (40 *μ*m thick), supposing the simultaneous presence of the resin in both of the layers.

**Figure 10:**
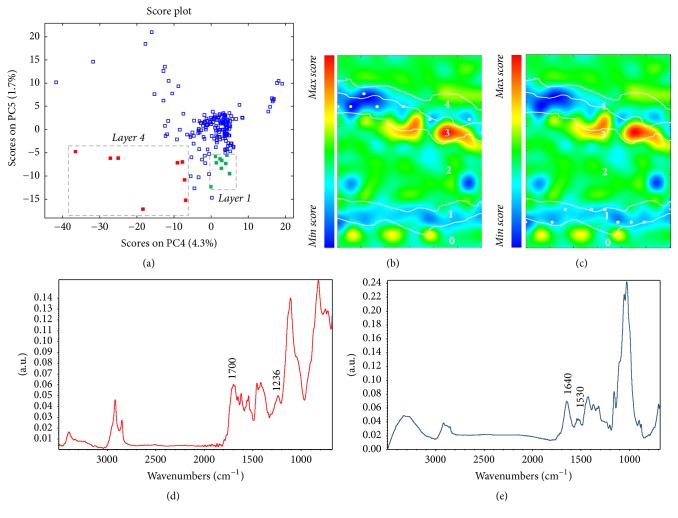
Sample BC1—(a) PC45 score plot: cluster highlighted in red indicates the objects points localised within layer 4 (white dots in (b)) and cluster highlighted in green indicates the objects in layer 1 (white dots in (c)); (b and c) PC5 score maps; (d) average spectral profile extracted from layer 4; (e) average spectral profile extracted from layer 1.

Multivariate analysis was applied to better describe the organic layers present within the stratigraphy. In particular, the PC1 score map* (data not shown)* allowed the differentiation among the KBr embedding system, cellulose, and all of the other components in the sample area submitted to analysis. Moreover, lower-order principal components were examined in attempt to obtain more detailed information. Thus, it was possible to clearly recognise the two organic layers in PC5 score map (Figure 9(b)). The joint analysis of the corresponding score scatter plot, as well as the extraction of the average spectrum from each layer, permitted the identification of the IR spectral features most involved in the molecular characterisation of the layers. In the PC4 versus PC5 score plot (Figures 10(a) and 10(b)), two different groups of points are shown. In particular, for the groups of points (red squares) which are characterised by a mid-score value along PC4 and a mid-low value along PC5, a good correspondence to layer 1 is noticeable. The loading plot shows the role of the band at 1230 cm^−1^ (C–O stretching) that can be ascribed to a natural resin* (data not shown)*; such an absorption band in layer 4 is also visible in the extracted spectrum (Figure 10(d)).

Moreover, the green squares that present a mid-high score value along PC4 and low value along PC5 clearly matched with layer 1. Spectral interpretation of multivariate data allowed the recognition of the contribution ascribable to the band at about 1540 cm^−1^ (amide II), and the extracted spectra (Figure 10(e)) suggested the presence of a proteinaceous component in the above-mentioned layer, together with some traces of calcium carbonate. Moreover, the presence of proteins may be also corroborated by the bluish fluorescence colour well localisable in layer 1 under UV illumination. Interestingly, with the univariate approach, the contribution of both of the components could not be identified, disclosing new information on the execution technique adopted by Vincenzo Coronelli.

## 4. Conclusions

The proposed approach, based on the well-known potentialities of multivariate analysis, allows an efficient spatial stratigraphic localisation of compounds of interest, as well as an efficient differentiation between heterogeneous mixtures characterised by similar spectral features, that could not be identified by univariate studies (single-band based maps). In fact, up to now, only few examples were reported in the literature on the application of multivariate mapping for the study of paint cross-sections and this research reports and proposes a systematic strategy which implements a combined application of *μ*ATR-FTIR mapping and chemometric analysis. In particular, the multivariate methodology applied proved to be a powerful and efficient tool for extracting valuable information from map spectra, definitely suitable for a straightforward interpretation of complex matrices such as paint cross-sections.

Several chemometric tools (e.g., brushing, for the localisation of scores in PC maps, and the extraction of average spectral profiles) were employed for a complete extraction of the useful information contained within the original data. Furthermore, all the information highlighted by the most representative PC score maps was combined in single false colour images, thus obtaining a comprehensive chemical mapping. The methodology presented represents a fast and not expensive exploratory procedure that can be applied for the interpretation of *μ*ATR-FTIR chemical maps, avoiding the loss of important amounts of the global information embodied in the hyperspectral datasets.
